# Supplementary material for: Are carbon emissions trading and green financial instruments synergistic? -Comprehensive quantitative research based on content analysis
Source: PLoS One. 2024 Mar 7;19(3):e0298601. doi: 10.1371/journal.pone.0298601 (PMC10919601; doi:10.1371/journal.pone.0298601)
Supplement: S1 Data — (ZIP) [file pone.0298601.s001.zip › Data/地级市Z_F工作报告.zip!/─■╧─/╓╨╬└╩╨/└┤╫╘╣1⁄2╓┌║┼í╛┬φ┐╦╩2╛▌═°í┐_2012.docx]

各位代表：

现在，我代表市人民政府，向大会作政府工作报告，请予审议，并请各位政协委员和列席人员提出意见。

2011年工作回顾

2011年，是实施新一轮西部大开发战略和“十二五”规划的起步之年，也是我市经济社会发展取得显著成绩的一年。在自治区党委、政府和市委的正确领导下，在市人大、政协的监督支持下，全市上下深入贯彻科学发展观，认真落实市委二届七次、八次全体（扩大）会议精神，紧盯市二届人大四次会议确定的八个方面重点工作，以提升战略、注重创新、提高能力、狠抓落实为关键环节，只争朝夕、开拓进取，全面完成了各项任务，经济社会呈现出更好更快的发展态势，实现了“十二五”开好局、起好步的目标。预计全年地区生产总值突破200亿元，达到213.6亿元，增长13.5%；完成固定资产投资194亿元，增长25%；地方财政收入突破20亿元，达到21.7亿元，增长33.9%。其中，一般预算收入完成12.1亿元，增长42.4%，创历史新高；城镇居民人均可支配收入达到15800元，增长13%；农民人均纯收入达到5060元，增长14%。

一年来，主要做了以下六个方面的工作：

一、紧紧围绕经济发展主导产业，突出发展工业

工业经济贡献凸显。预计完成工业总产值268亿元，增长54.8%；实现工业增加值69.8亿元，增长26.1%。工业化率提高到32.7%，工业经济对GDP的贡献率达到56.6%。建成工业项目15个，主要是：瑞泰精细化工、金阳银阳光伏产业、华夏特钢镍铁、天元锰业金属锰等；在建项目18个，主要是：渝丰化工、锦宁巨科铝板带箔、茂原黄腐植酸等。新能源、新材料、冶金、化工、造纸、农副产品深加工6大优势特色产业不断壮大。宁夏钢铁、锦宁铝镁、中冶美利纸业、隆基硅材料等大型龙头骨干企业规模效益进一步提升，带动能力明显增强。

招商引资成效显著。全市招商引资签约落地项目61个，总投资1057亿元，总产能1258亿元。主要是：投资373亿元的广汇申能能源及化工，投资122亿元的酒钢集团特钢，投资116亿元的中冶集团“长江项目”，投资26亿元的上海亿丰集团城市商贸综合体，投资176.5亿元的银阳硅业多晶硅、光伏制造及金阳公司光伏发电，投资38.8亿元的锦宁铝镁合金二期，投资30亿元的华电集团风力发电及投资5.6亿元的华创风机制造等。到位资金166.5亿元，增长20%，超额完成自治区下达的115亿元任务指标。全力打造“三个千亿元产值”工业园区，不断完善“三区六园”基础设施，中卫工业园区向东扩展20平方公里，全市工业园区建成面积达95.5平方公里。

节能减排有新成效。严格执行节能目标责任制和十大节能减排铁律，坚持上大压小，优化结构，共淘汰落后产能74万吨，累计节约能源消费量65万吨标准煤。组织实施节能项目，大力发展循环经济和清洁生产。取缔查封了所有违法违规矿点和小陶瓷窑。实施中冶美利纸业氧化塘防渗漏等重点工程。全市二氧化硫、氮氧化物、化学需氧量、氨氮分别削减6021吨、3544吨、4379吨和434吨，完成了自治区下达的减排任务。

二、紧紧围绕农业增效农民增收，坚持不懈做好“三农”工作

农业经济稳步发展。预计全年实现农业增加值39.7亿元，增长5.5%。全面落实各项惠农政策，粮食播种面积257万亩，总产量5.4亿公斤，增长3.1%。全市硒砂瓜、枸杞、设施蔬菜、马铃薯种植面积分别达到90.5万亩、37.6万亩、27万亩和111.7万亩。鸡、猪、牛、羊饲养量分别达到986.1万只、103.7万头、26.4万头和173.8万只。实施永大线高效节水项目二期、南华山水源涵养林、中卫工业园区等绿化造林工程，完成植树造林43.4万亩。狠抓禁牧封育工作，偷牧行为得到有效遏制。

特色产业提质增效。优势特色产业对农民现金收入的贡献率达到29%。瞄准高端市场，加快农村土地流转，建设供港蔬菜基地2.2万亩，比较效益明显。中宁县新建出口枸杞基地1.1万亩，海原县新建马铃薯脱毒一级种薯生产基地2万亩。兴仁镇兴仁村硒砂瓜、宣和镇宣和村蛋鸡、舟塔乡康滩村枸杞、高崖乡草场村西甜瓜入选“全国一村一品示范村镇”。

基础设施不断完善。对集中连片的农村危房改造示范点和“塞上农民新居”示范点，实施“五化、四改、三通”工程。建设文体休闲广场17个，硬化道路300公里，铺设排水管道110公里，村庄植树18.9万株，清理“三堆”32.8万立方米，拆除棚圈13.7万平方米。设立水利发展专项资金1000万元，加大农田水利基础设施建设力度，完成兴仁综合供水工程续建、基本农田保护示范区建设、海原园河库井灌区改造等重点水利工程。全面实施宣和、兴仁等重大土地整理项目，整理土地8.6万亩。

生态移民成效显著。建成沙坡头区香山米粮川和兴仁高庄、王团生态移民项目区，迁入移民2039户9130人。基本建成沙坡头区康乐、敬农，中宁宽口井，海原海城等11个项目区和沙坡头区8个分散移民点，建设住房5646套。因地制宜制定移民区产业发展规划，大力推进枸杞、覆膜滴灌马铃薯和肉牛繁育为主的移民后续产业，实施“公司+基地+农户”发展模式，使移民人均纯收入达到4000元以上。

三、紧紧围绕旅游带动服务业，第三产业增添新活力

旅游产业实现突破。引入战略投资，与旅游业龙头企业香港中旅集团签订了合资合作协议，对完善沙坡头景区旅游设施、提高经营管理水平、扩大经营规模、延伸产业链条、拓展品牌效应将产生深远的影响。加大旅游基础设施建设力度，建成金沙岛旅游休闲度假区二期等工程，完成投资1.9亿元，接待能力进一步提高。启动实施应理水镇、黄河祁连山大峡谷疏浚工程，组织实施旅游营销宣传“十个一”工程，提升了中卫旅游的知名度。全年接待中外游客190万人次，增长22.6%，旅游总收入达到14亿元，增长40%。

交通物流商贸加快发展。编制完成《迎水桥大西北物流城概念性规划》，基本完成了迎水桥大西北物流城、全民创业城、城市商贸综合体土地房屋征收工作。香山机场整体移交西部机场集团。全年新建改建公路560公里。落实“万村千乡市场工程”，大力推行“农超对接”，实施“家电下乡”惠民工程，农村家庭享受国家政策补助4900万元。预计全社会消费品零售总额达38.4亿元，增长17.4%。外贸进出口总额达1.5亿美元，增长5.4倍。

新型服务业拓展迅速。大力发展城市社区服务、家政服务等生活性服务业，从业人员达4200人。全市新增酒店36家、床位3200张。扩大保险服务业的广度和深度，全年保费收入达4亿元。挂牌成立了中卫农村商业银行和香山村镇银行，积极发展信用担保中心等新型金融服务业态，预计全市存款余额233亿元，贷款余额208亿元，新增贷款48亿元，比上年净增16.8亿元，金融对经济的支持力度进一步加大。

四、紧紧围绕统筹城乡发展，城镇化步伐明显加快

城市化明显加快。以“五创”为重点，高标准规划、建设和管理城市。完成《中卫市城市总体规划（2011-2030）》修编，城市规划区面积由51.5平方公里调整到67.5平方公里，控制面积由400平方公里调整到2260平方公里，城市建成区面积32平方公里。全年实施城市重点建设项目87个，完成投资45.5亿元。开工建设房地产项目13个，续建41个，建筑施工面积296万平方米，商品房销售面积115万平方米。发放住房公积金贷款1.5亿元。全市城镇化率达到31.9%，提高2.4个百分点。其中沙坡头区达到45.2%，提高2.9个百分点。

城市价值大幅提升。大力实施城市植绿、增绿、扩绿工程，完成城市绿化1.9万亩，栽植各类苗木1170万株，新增城市绿地面积519万平方米，绿地总面积达到1637万平方米。沙坡头区绿化率达到39.1%，绿地率达到36.6%。精心实施引水、增水、扩水工程，水域面积达到3万亩。利用腾格里沙漠丰富渗水资源，巧妙连通六大水域，城市水系四季流淌、水面饱满、晶莹剔透，使城市充满了灵动和活力。通过绿化、水系和公共基础设施建设，大大提升了城市品位和价值，市区土地均价达到每亩60万元。

城乡面貌显著改善。建成集体育馆、文化馆、图书馆、游泳馆、博物馆和文体娱乐中心于一体的市“五馆一中心”，完成应理路等12条旧街区和“城中村”改造。加强对环境卫生、园林绿化、公园广场等的管理，全面推进“三大工程”及铁路沿线环境整治工程，建成“塞上农民新居”示范点9个，危房改造示范点14个，环境综合整治示范村庄57个，改造农村危房14734户。实施了沙坡头区宣和、镇罗、中宁县石空等小城镇改造工程。

五、紧紧围绕构建和谐社会，民生民利得到保障和改善

科技教育事业有进步。支持科技型中小企业，新培育科技型中小企业6家，专利授权18件。引进推广农业新品种20个，种养业良种覆盖率达到95%。坚持优先发展教育，建成中卫六中、八小、海原兴海中学，完成139所学校23.7万平方米的校安工程，基本解决了大班额、大通铺及校舍安全问题。海原县通过自治区教育强县评估验收。中卫职业技术学校被确定为国家中等职业教育改革发展示范学校。实施贫困地区农村学校小学生免费午餐试点工程。加快发展学前教育，改扩建幼儿园10所。加强教师队伍建设，教学质量有所提升，全市高考录取率达75.4%，提高4.7个百分点。

文化卫生事业健康发展。举办了中卫市首届元宵灯展暨美食节，新建农家书屋309个，举行广场文艺、红歌传唱、民风建设等文艺演出153场，《回乡婚礼》累计演出600余场。新建2个非物质文化遗产传承保护基地，完成第三次全国文物普查。《武动的响板》获第九届全国少数民族传统体育运动会表演项目一等奖。建成市中医医院康复楼、沙坡头区人民医院门诊综合楼、中宁县医院住院部和自治区人民医院宁南医院。完善远程医疗会诊平台，建立并实施药品来源电子化监管、餐饮服务食品安全标准与监督管理长效机制。全市人口出生率、自然增长率分别控制在12.25‰和8.66‰以内。

社会保障水平逐步提高。在全区率先建立农民工工资“一卡通”直接支付制度，从源头上解决了建筑领域农民工工资拖欠问题。建立农民工市民化户籍制度和社会保障制度，解决农民工被城市边缘化问题。全面启动统筹城乡居民的社会养老、医疗保险工作。为720名重特大病患者发放救助金1780万元，为8214户困难家庭发放廉租住房补贴3057万元，为2638户提供了廉租住房。率先实施重度残疾人社会保障制度，建成中卫市儿童福利院，为环卫绿化工人提高了工资标准。建立中卫市创业互助基金，创办小企业391个，培养小企业主394个，新增就业岗位3752个。

6件民生实事全面落实。建立新市民保障体系，为8972名农民工办理“农转非”；沙坡头区开工建设保障性住房56.7万平方米，主体完工34万平方米；实施新型生态移民工程，建成住房4329套，兴仁移民区安置入住1776户，教育培训移民558人；实施创建“中国人居环境奖”城市旧街区“四化”工程，改造应理路、长城街、文昌街等12条14公里旧街区，完成“城中村”改造8处3268户；开工建设中卫职业技术学校二期工程和市救护康复体检中心；建成全民创业慈善工业园一期44栋标准化厂房。

六、紧紧围绕效能建设，体制机制不断创新

社会管理不断创新。深入开展“建设和谐富裕新中卫大讨论大走访大调研”活动，排查调处矛盾纠纷5192件，优良民风正在形成。成立涉法涉诉联合接访服务中心，制定实施中卫市信访受理处理督办考核维序五联动、信访包案责任制、终结制等制度，化解重点信访积案40件，进京非正常访和到自治区集体访分别下降31.6%和48.9%。完善“三调联动”机制，全市调处各类矛盾纠纷14502件。建立行业性专业化人民调解工作机制，调解交通事故损害赔偿、医疗疑难纠纷891件。加大法律援助力度，办理法律援助案件2100余件。率先建立安全生产责任网格监管体系，全市安全生产事故死亡人数比自治区下达的控制人数减少21人。建立治安巡防和道路交通安全巡查制度，城市重点部位15分钟、县乡道路30分钟间隔巡逻，治理道路交通黑点474处，查处违法行为6041起，道路交通死亡人数下降32.7%。

政府项目资金监管有力。率先建立政府投资项目造价与发包分离制度，实行包干造价，共审定招标书120个，节约资金8000余万元，探索出了一条节约投资、提高效益、有效防止工程建设领域滋生腐败的新路子。出台《加强财政科学化精细化管理的意见》，全面推行财政资金科学化、精细化管理。强化行政监察和审计监督，开展了工程建设领域突出问题、公务用车和“小金库”专项治理。

政府效能持续提升。认真执行《中卫市人民政府廉政效能建设九项制度》，推行市长督办副市长重点工作制度，实施重点工作“五定”工作法，市政府常务会议督办重点工作318项，有效推动了工作落实。实行政务服务中心八项工作制度，办结各类事项45075件。建立市长信箱岗位工作九项规定，解决群众合理诉求2849件。全面改版政府网站，建立法制网站，信息点击量大幅提升。切实实施《中卫市国家工作人员法律与专业三年培训规划》，组织培训24场、考试528场2.3万人次，国家公职人员履职意识和能力增强。

依法行政不断加强。认真执行市人大及其常委会各项决议，坚持向市政协通报工作，密切联系各民主党派和群众团体，自觉接受社会各界监督。共办理人大议案、建议49件，政协提案227件，办复率100%。全面贯彻依法行政实施纲要，“六五”普法工作全面启动。坚持科学、民主、依法决策，加强行政执法行为监督检查，切实纠正行政执法违法行为。深入开展“命案侦破”、“打黑除恶”，打击“两抢一盗”、经济犯罪和“传销”等专项行动。百名民警破案数达152起，命案全部侦破，社会治安持续好转。

全面贯彻党的民族宗教政策，各族人民和谐相处。扎实开展双拥共建，中卫市荣获“自治区双拥模范城”称号。建成民兵训练基地和应急救援队伍，经常性开展国防教育和地震、消防、交通安全等应急演练，人民群众的国防意识、抗震减灾意识进一步增强。民兵预备役、人民防空、统计、地方志、档案、外事、侨务、保密、老龄、残疾人、邮政、通信、气象等各项工作都取得了新成绩。

各位代表！

发展的历程令人难忘，发展的成就令人欣慰，发展的势头令人振奋，发展的愿景令人向往。这些成绩的取得，是自治区党委、政府和市委总揽全局、正确领导的结果，是市人大、政协支持配合、有效监督的结果，是全市广大干部群众真抓实干、负重拼搏的结果，是社会各界凝心聚智、和衷共济的结果。在此，我代表市人民政府，向全市人民，向人大代表、政协委员，向各民主党派、工商联、无党派人士、人民团体，向驻卫部队、武警官兵，向所有关心、支持和参与中卫建设与发展的同志们、朋友们，表示崇高的敬意和衷心的感谢！

在充分肯定成绩的同时，我们也要清醒地看到发展中存在的困难和问题：一是经济总量较小，人均水平较低，城乡差距较大；二是因能耗刚性约束，大大增加了招商难度；三是“三农”问题依然突出，农村经济薄弱，农民持续增收困难较大；四是社会组织发展滞后，社会保障等民生方面还存在一些亟待解决的问题，社会维稳难度加大；五是政府工作人员执行力还不够强，行政效能还需进一步提高。上述困难和问题，我们将认真研究，在2012年的工作中切实加以解决。

2012年重点工作

2012年，是全面实施“十二五”规划、建设和谐富裕新中卫承上启下的关键之年。世界经济复苏的不稳定性、不确定性上升，我国经济面临出口明显下降、物价上涨、经济增速趋缓。稳增长、调结构、控通胀将成为明年经济工作的主基调，国家将实施积极的财政政策和稳健的货币政策，进一步加大对“三农”、重点项目、创新型项目、保障性安居工程和微型小型企业发展的政策扶持力度，为我市加快发展创造了有利的外部条件。刚刚胜利闭幕的中共中卫市第三次党代会，指明了中卫未来发展的方向和目标，找到了实现科学发展、创新发展、跨越发展的战略路径，形成了科学发展、创新发展、跨越发展的整体合力。只要我们坚定信心，“眼里有活、用心做事、敢于担当、真心负责”，就一定能够在建设和谐富裕新中卫的宏伟征程中阔步前进！

政府工作总体要求是：以邓小平理论和“三个代表”重要思想为指导，深入贯彻科学发展观，认真落实自治区党委十届十四次全体（扩大）会议、全区经济工作会议精神和中卫市委二届八次全体（扩大）会议、第三次党代会精神，以“疆煤进宁、旅游振兴、城市化带动”三大战略和“创新引领、特色产业倍增、民生改善”三项计划为行动纲领，着力培育产业发展新优势，着力打造宜居宜业新环境，着力满足人民幸福生活新期待，奋力开创建设和谐富裕新中卫的崭新局面。

主要预期目标和约束性指标是：完成地区生产总值247亿元，增长12%；完成全社会固定资产投资250亿元，增长25%；地方财政一般预算收入完成14亿元，增长15.7%；城镇居民人均可支配收入达到17696元，增长12%；农民人均纯收入达到5670元，增长12%；社会消费品零售总额实现44.5亿元，增长16%；城镇新增就业7550人，城镇登记失业率控制在4.2%以内；人口自然增长率控制在10.4‰以内；力争完成自治区下达的节能减排任务。

实现上述目标，重点抓好以下六个方面工作：

一、加快推进现代农业，以提高“双率”实现农业增效农民增收

始终把“三农”工作摆在重中之重的位置，以提高土地产出率和劳动生产率为核心，调整农业产业结构，转变发展方式，着力将传统农业改造成为高效生态现代农业，力促农业增效、农民增收、农村繁荣。完成农业增加值44.3亿元，增长5.5%。

着力发展现代农业。大力实施设施农业提质增效工程，建成10个设施农业提质增效示范区；积极推广“公司+基地+农工+高端市场”发展模式，新建供港蔬菜基地1万亩，建成万亩蔬菜及花卉制种基地、万亩稻田生态种养基地、2万亩立体生态高效渔业基地，培育沿黄灌区设施农业集约化产业带；大力发展经济林，新增苹果、枸杞、红枣面积1.2万亩、4.1万亩、0.6万亩，培育扬黄灌区经济林产业带；加快发展畜牧业，建成万头现代化奶牛生态观光养殖牧场、4万套种鸡孵化场和10个生态移民区肉牛养殖示范村，培育中部干旱带草畜产业带。积极扶持夏华清真牛羊肉、万盛枸杞等农副产品龙头企业做大做强。开工建设沙坡头区农产品批发市场，壮大农村经济组织、经纪人队伍，培育年销售过亿元流通企业和流通组织5家。实施品牌带动战略，加强“中宁枸杞”、“香山硒砂瓜”等品牌保护，实现特色农产品由“卖产品”向“卖品牌”转变。

加大农业基础设施投入。开工建设沙坡头南北干渠改造工程，完成南山台扬水泵站改造、涩井沟治理和黄河防洪等水利工程。实施香山米粮川二期、敬农生态移民区、常乐项目区等重大土地整理项目。坚持不懈地开展农田水利基本建设，大力改造中低产田，积极推进水利改革和水权转换。重点抓好南华山水源涵养林基地、中卫滨河沙漠湿地保护示范工程、长流水荒漠化治理、农田林网等植树造林工程，不断夯实农业发展基础。

着力推进新农村建设。将经营管理城市的理念引入农村，以农村环境综合整治“三大工程”为抓手，进一步改善农村环境面貌。新建“塞上农民新居”、农村危房改造示范点12个，综合整治旧村庄33个。落实农村环境综合整治长效机制，彻底改变农村脏乱差现象。改革优化乡镇职能，切实发挥民生服务中心作用，加强村民自治，壮大村级集体经济，提高服务和保障能力，扎实推进新农村建设。

积极实施扶贫开发和生态移民。抓住国家新一轮扶贫开发机遇，创新理念，制定新的扶贫开发规划，以生态移民工程为主，变“救济输入”型为“扶智扶力”型扶贫，从根本上解决贫困问题。确保明年8月底前沙坡头区康乐、敬农，中宁宽口井，海原海城等11个生态移民项目区搬迁，入住移民5646户2.48万人。开工建设敬农项目区二期、龙湖项目区、中宁县公租房、海原县山门二期等生态移民项目工程，新建移民住房1230套，安置移民5400人。编制《中卫市慈善城市创建规划》，实施“黄河善谷”战略，成立中卫市公益慈善基金会，把海原工业物流园区建成慈善产业开发区。组建中卫市生态移民产业发展小额贷款公司，扶持产业发展，确保移民搬得出、稳得住、有产业、能致富。

二、加快推进疆煤进宁战略，以新型工业化主导发展

充分依靠工业在经济发展中的核心带动作用，着力提高工业比重，利用交通、土地、水利、区位组合因素形成的差异化优势，把实施疆煤进宁战略作为实现经济跨越发展的主要路径，大力发展新型工业。完成工业增加值83.8亿元，增长20%。

狠抓工业园区基础设施建设。力促建设疆煤进宁项目重大基础设施，主要是甘武铁路复线、新疆红柳河至淖毛湖铁路专线，开工建设中卫工业园区铁路专线。完成中卫工业园区东扩区20平方公里市政绿化工程，完善工业园区一期工程基础设施。提升完善中宁工业园区规划，开工建设跨包兰铁路高架桥。进一步完善海原工业物流园区基础设施，开工建设污水处理厂。充分利用自治区赋予的国有荒地新增规费支持工业发展优惠政策，一次规划、逐年建设，逐步实现中卫工业园区与中宁工业园区一体化，形成350平方公里的全区重大的工业集群地、产业承接地和经济发展极。

狠抓重大项目推进。狠抓27个在建项目建成投产，新增工业产值40亿元。主要是渝丰化工15万吨合成氨、蓝丰2万吨邻苯二胺、锦宁巨科40万吨铝板带箔、中电投1000兆瓦风电等项目。狠抓15个建成项目投产达效，完成工业产值68亿元。主要是银阳太阳能电池组件、宇光焦镁联产、华御一期、瑞泰一期、华业特钢金属锰、华电集团风电一期等项目。狠抓广申等25个重大项目开工，当年完成投资105亿元。主要是广申能源及化工一期1500万吨煤炭物流集散中心、2×100万千瓦超超临界空冷发电机组、2×5万立方米液化天然气转运分销中心、8座液化天然气加注站项目，宁钢320万吨特钢、中冶纸业“长江项目”、金阳公司200兆瓦光伏发电、银阳硅业5000吨多晶硅、华夏特钢10万吨锰酸锂、佳盛远达62万吨铝镁合金、华创风能1000兆瓦风力发电等项目。

狠抓招商引资签约落地。实施大招商招大商，创新招商方式，瞄准世界500强、中国500强、行业500强企业和上市公司，围绕新能源、新材料、冶金、化工、造纸、农副产品深加工6大产业板块，注重上下游配套、产业链招商，优先引进技术型、成长型、环保型的大项目、好项目，特别是风电光电等降低单位能耗的项目。优化招商引资环境，继续推行市领导包保、一企一策、一站限时、一门式服务等措施，着力打造“亲商、重商、尊商、富商”的“服务高地”和“政策洼地”。全年达成意向项目20个，合同签约项目20个，落地开工项目25个，招商引资到位资金145亿元。

狠抓节能减排和环境保护。制定招商引资企业单位能耗准入标准，通过能耗低的增量产值降低全市单位能耗指标，腾出工业发展空间。严格落实节能减排措施，大力推行能源管理目标责任制，切实不断降低单位能耗。认真执行“三同时”制度，力争全市万元地区生产总值综合能耗下降2.86%，化学需氧量、氨氮、二氧化硫、氮氧化物4项指标控制在自治区下达指标以内。加强环境保护，开工建设中冶美利浆纸、天净天达、新华冶炼等一批余热发电项目。完成中冶美利纸业废水深度治理环保工程。倡导绿色循环低碳发展理念，提升生态文明水平，努力实现人与自然和谐共处。

三、加快推进旅游振兴战略，以旅游业提升第三产业

坚持高起点规划，高标准整合旅游资源，完善产业链条，推进中卫旅游由随意开发向规划引领转变、资源战略向品牌战略转变、景区时代向目的地城市转变、特色产业向支柱产业转变，以旅游业带动第三产业大发展。

实施旅游振兴战略。以建设西部特色休闲旅游度假城市和国家级、国际化的沙漠旅游目的地城市为目标，高标准编制完成《中卫市旅游总体规划》。加快推进与港中旅合作项目的实施，开工建设沙漠王朝酒店、沙漠自驾营地、黄河祁连山大峡谷漂流等精品旅游项目。完成沙漠生态园、南长滩党项民俗村等系列开发项目，着力打造“万里黄河第一漂、千年党项第一村、大漠边关体验地、地空穿越大本营”的旅游胜景。扎实推进旅游与文化相结合，实施“一套书、一本画册、一台精品剧目、一支高水平的文艺队伍、一批独特的人文景点和一个文化旅游发展基金”“六个一”工程。强化宣传营销，积极开拓北京、上海等航空旅游市场，争取开通加密节假日旅游专列，大幅提升沙坡头品牌影响力和中卫旅游竞争力。全市旅游人数突破230万人次，增长22%，旅游总收入突破18亿元，增长28.6%。

大力发展房地产业。落实促进房地产业发展政策，鼓励企业加大开发力度。全年新开工商品房100万平方米。全市建成13152套86.2万平方米保障住房。其中，沙坡头区5268套40.5万平方米、中宁县5884套35.7万平方米、海原县2000套10万平方米。全面推行物业管理基本服务规范，沙坡头区住宅小区50%达到三级以上服务水平，40%达到二级以上服务水平。开展涉农职工住房公积金贷款业务，支持翻建住房，繁荣房地产市场。

积极发展交通物流现代服务业。树立大物流服务大产业、撬动大发展的理念，依托公路、铁路、航空等交通优势，构筑大物流，发展大商贸。争取立项建设包兰二线、宁夏西线高速、海原工业物流园区至县城一级公路等项目。扩建沙坡头区、中宁县两座黄河公路大桥，新建中卫工业园区第三座即广申大道跨铁立交桥。新建改建农村公路350公里。完善中宁物流园区、海原工业物流园区服务功能，加快建设迎水桥大西北物流城、广汇煤炭物流园区。建设城市商贸综合体，培育并逐步做大小商品市场，辐射周边，繁荣商贸，带动加工产业。力争引入第三方物流企业，以此做大物流业。鼓励金融机构加大信贷投入，优化信贷结构，年新增贷款50亿元，支持地方经济发展。全年外贸进出口总额达到1.7亿美元。

四、加快推进城市化带动战略，以高定位获得城市未来发展优势

在工业化、城市化和现代化三重叠加的发展过程中，城市化是重中之重。始终坚持城市化质量决定未来经济高度的发展理念，始终坚持把“城市让生活更美好”作为城市化的最高战略，始终坚持把“宜居、休闲、生态美”作为城市化的最高定位，高水平建设经营管理城市，着力提升城市的价值度、聚集力和辐射力，让中卫在城市化过程中获得未来发展优势。

建设黄河金岸重大项目。按照自治区党委、政府把沿黄城市建成城市带、产业带、开放带的要求，加快实施黄河金岸重大项目。全市开工建设77个黄河金岸重大项目，总投资1162亿元。主要是黄河生态运动休闲公园改造提升、广申能源及化工、锦宁铝镁合金二期、应理水镇、城市商贸综合体、迎水桥大西北物流城等项目，当年完成投资206亿元。

加快推进城市化。实施老城区环境提升工程，改造"城中村"2000户，完成市区20条街道“四化”、情怀河亮化和利民路、西一路等6条城市道路工程。建成中宁县宁安西街延伸段、宁丰路北段、新区富康东路等8条道路，实施海原县城道路、西门市场改扩建、城市绿化等工程。完善城市公共交通规划，成立公交公司，开通部分公交线路。建设中卫黄河水厂、第二中水厂和海原县中水厂。完成石空、宣和、镇罗、柔远等沿黄特色小城镇改造工程，积极发展餐饮、文化、商贸等服务业，增强城镇承载能力。解决好务工人员就业、廉租房保障、子女上学等突出问题，进一步放宽农民工户籍登记，促进农村剩余劳动力向中心集镇聚集转移。全市城镇化率提高到34%，沙坡头区达到48%，分别提高2.1个百分点和2.8个百分点。

切实提高城市管理水平。坚持建设与管理并重，理顺城市管理职能，持续开展市容市貌专项环境整治，下大力气解决人民群众反映强烈的占道经营、交通拥堵、乱停乱放、“如厕难”等突出问题。严格落实门前“一定五包”责任制，广泛吸纳社会力量参与环境卫生管理。深入推进创建国家园林城市、卫生城市、环保模范城市、中国旅游目的地城市和全国文明城市“五创”工作，构建优美和谐人居环境。启动数字城市建设，提升城市管理水平。

五、加快推进社会各项事业，以改善民生构建和谐

牢固树立人民至上的理念，持续推进社会各项事业和民生计划，将更多的财力向民生倾斜，让发展成果更多地惠及广大人民群众，不断提升人民群众的幸福感。

办好15件民生实事。实施沙坡头区饮水全指标检测和水质改造工程，彻底解决群众饮水安全问题。实施中卫职业技术学校改造提升工程。着力提高学龄前儿童入园率，建成10所幼儿园。实施贫困地区农村学校小学生免费午餐工程。建设市救护康复体检中心。全面开放一批重要文化体育设施。完成农村直播卫星公共服务户户通工程。实施“送欢乐下基层”文艺演出活动。实施黄河生态运动休闲公园提升工程。建设保障性安居工程。继续实施生态移民工程。实施老年人安康保障工程。创新社区建设与管理新机制。实施重度残疾人生活津贴制度。全面统筹城乡居民养老保险和医疗保险工作。

加快发展教育科技事业。优化教育结构，合理配置资源。加快发展学前教育，推进义务教育均衡发展，加强师资培训，实施素质教育。完成中卫职业技术学校提升工程，为升格高职学院打好基础。新建中卫市第九小学、中宁县特殊教育学校，建成10所幼儿园，使三年期幼儿园孩子比例从22.9%提高到28.9%，逐步解决学龄前儿童“入园难”问题。实施贫困地区农村学校小学生免费午餐工程，改善全市贫困地区425所学校7.3万名小学生的营养健康状况。加快科技推广转化利用，注重引进和培养高素质专业人才队伍。建成国家、自治区级企业工程技术中心和技术实验室5个，培育科技型中小企业8家、企业技术中心6家。

大力发展医疗卫生事业。建成市救护康复体检中心，填补中卫没有康复、体检和危重病症救护中心的空白。新建50所标准化村卫生室，切实改善群众就医条件和村医待遇。深化医药卫生体制改革，有效缓解“看病难、看病贵、看不好病”问题。建立城乡统一的居民电子健康档案管理系统，实现全市医疗信息查询收录互联互通。做好重大疫病防控工作，提高应对突发公共卫生事件能力。强化计划生育国策地位，建立流动人口计划生育社区即时跟踪管理模式，严格落实孕情监测报告及处理例会等监管措施，切实稳定低生育水平。

力促文化大发展大繁荣。按照自治区把文化旅游产业培育成重要支柱产业和新的增长点的要求，加大投入，加快推进文化旅游融合发展。开工建设万达城市文化综合体，开放使用市“五馆一中心”，免费开放市、县文化馆、图书馆、博物馆，加强乡镇文化站、村文化活动室和农家书屋建设管理，丰富城乡百姓业余文化生活。完成22万户直播卫星公共服务户户通工程，确保收视28套电视频道。实施文化下乡惠民工程，组织文艺团队进乡村、进社区、进学校，演出760场，农村数字电影放映7500场以上，使每个行政村、社区、学校都能看到文艺演出，每月都能看到一场电影。加强大麦地岩画等文化遗产保护和开发利用，积极推进刺绣、剪纸等民间非物质文化遗产市场化发展。创新文化人才培养、引进和管理机制，鼓励多出、快出、出好作品。大力发展文化产业，着力打造《印象中卫》等精品剧目，积极拓展新的文化业态，提升中卫文化软实力。

全面促进社会和谐稳定。以建设社会主义核心价值体系为根本，以思想道德建设为重点，着力实施“法制教育、文明新风培育、城乡环境整治、基层民主管理”四大工程，深入推进民风建设。启动中卫市五联动综合信访接待程序，继续实施领导包案责任制，积极化解社会矛盾纠纷。广泛开展群众性精神文明、民族团结、国防教育、双拥共建等活动，依法管理宗教事务。强化安全生产管理，认真落实安全生产“三同时”、“三排查”和“一岗双责”制度，杜绝重特大生产安全事故发生。深化“平安中卫”创建活动，继续开展“打黑除恶”、打击“两抢一盗”、涉众型经济犯罪等专项整治活动。扩大市区治安联防范围，严厉打击各类违法犯罪活动，努力构建和谐稳定的良好社会环境。

六、加快推进体制机制改革，以创新引领科学发展

以永不衰竭的热情和不断迸发的激情，深入改革、着力创新，摒弃习惯做法，突破思维定势，不断打破体制束缚，不断释放活力动力，不断获得倍增效益，不断推进跨越发展。

大力推进5项改革。改革沙坡头区行政管理体制。理顺市、区职责关系，科学合理划分事权，强化乡镇和社区职能，建立职责明晰、管理有序、精干高效的管理体制。分类推进事业单位改革。开展事业单位评估工作，按照承担行政职能、从事公益服务和生产经营活动三种类别，清理规范事业单位，形成基本服务优先、供给水平适度、布局结构合理、服务公平公正的公益服务体系。建立政府投资项目单位工程预算标准制度。通过每个分项单位工程造价标准化，有效控制工程造价，大幅降低建设成本，节约资金。建立公务卡消费制度。预算部门公务消费全部实行公务卡结算，明确强制结算目录，实现公务消费公开、透明、真实，有效防止“三公”腐败，为细化部门预算提供核定依据。建立节余专项资金统筹使用制度。把中央、自治区专项投资项目，纳入造价与发包分离制度和单位工程预算标准制度进行管理，科学有效地控制造价，对节约的专项资金实行分类统筹、集中使用，切实提高财政资金使用效益。

实施10项创新工作。完成中卫发展战略规划研究与制定。聘请国内权威战略研究机构和知名专家，在国家和区域战略布局中，找准中卫跨越发展的战略与路径，实施“三大战略”，把中卫交通、水利、土地和区位形成的组合优势转化成经济发展优势。建立小型微型企业个体工商户大发展新机制。

落实国家关于引导、鼓励和支持个体、私营等非公有制经济发展的政策措施，把小型微型企业、个体工商户作为经济活力的源泉，重点扶持。鼓励下岗职工、困难家庭、大学生、农民工等创业就业，有效壮大民营经济，为发展注入新活力、新动力，培育新增长点。创新招商引资机制和模式。实行国有荒地新增规费支持工业发展优惠政策；探索直供电模式；实行招商项目万元增加值能耗指标准入政策，形成政策效应，招大商招好商，加快工业发展。全面实施信访五联动和信访终结制度。推行“一站式接访、一条龙服务、一揽子解决”信访工作模式，建立久访不息信访事项的终结机制，依法维护信访秩序，实现有序理性上访，维护法律权威和公平正义。深化医药卫生体制改革。克服体制积弊，提升市、县医院诊疗水平，有效缓解危重急症和疑难病症诊疗难题；创新基本药物供应方式，有效缓解“看病贵”问题；严格科学诊疗程序，有效缓解“看不好病”问题。实施硒砂瓜品牌保护和提质增效工程。界定并保护香山硒砂瓜产地；严格实施标准化生产技术规程，保持独有口味并不断提高其品质；严格品牌认证与保护，严厉打击各类侵权违法行为；积极拓展市场，加强营销管理，实现硒砂瓜产业健康持续发展。建立社区管理新体制。在中山、东方红、民族巷社区开展改革试点，探索新形势下现代社区管理新模式。适应城乡一体化建设的需要，推进无土地村（组）管理向居民委员会管理模式转变。建立校车安全有效运营与安全即时监管机制。建立政府补助、市场化运营的校车运管新模式，由专业客运公司承担学生和幼儿接送任务；建立校车路查路巡、违法举报等制度，严厉惩戒校车违法行为。建立消防安全即时监管机制。对全市人口密集场所实行即时监管，严格惩戒违法违规者；建立消防安全信息平台，每天掌握重点消防单位火灾隐患动态，第一时间消除消防安全隐患。建立餐饮卫生安全监管无遗漏机制。对餐饮服务单位进行长期性、全覆盖、不间断的达标监督检查，有效保障餐饮食品安全。

着力优化发展环境。深入开展进一步营造风清气正的发展环境活动，规范执法行为，改革服务方式，提高服务水平，严肃查处不作为、慢作为、乱作为等损害经济发展环境的行为，以优化中小企业发展环境为重点，营造勤廉高效、服务优良的政务环境，公平正义、执法为民的法制环境，诚实守信、尊重规则的人文环境，规范有序、公平竞争的市场环境，灵活便捷、服务发展的金融环境，公正民主、风清气正的用人环境。

不断加强自身建设。主动接受人大及其常委会的法律监督和政协的民主监督，密切联系各民主党派和群众团体，自觉接受社会各界监督。认真办理人大议案、建议和政协提案，做到件件有着落、条条有办复。制定更具工具属性的公职人员考核办法，着重考核业绩、出勤、培训、专业四项内容，建立手机考勤终端信息平台、公职人员日志制度、专业培训档案、专业水平考核标准，以制度激发公职人员干事创业的热情和活力。严格执行行政监察办法，对重点工作两次督办逾期未完成的，进行督询，督询后仍未完成的，由监察部门提出行政监察建议，按规定严肃处理。严格执行“五定”工作法、市长督办副市长等层层督办落实机制，努力建设忠诚事业、精通业务、作风优良、诚实守信的国家公职人员队伍。加强党风廉政建设，严格执行党风廉政建设各项规定，严厉查处“小金库”和公务消费、公务用车等违纪违规行为。认真执行造价与发包分离制度，加强对土地出让、征地拆迁、采矿权交易、生态移民、禁牧封育等工作的监督检查。大力弘扬艰苦奋斗的作风，精简会议和文件，严格控制庆典活动和公务接待标准，努力降低行政成本，把更多的资金用在促进发展、改善民生上，让改革成果惠及全市人民。

各位代表！

建设经济快速增长、民生显著改善、社会和谐稳定、环境优美宜人、充满发展活力的新中卫，是时代的召唤，更是全市人民的热切期盼！让我们在自治区党委、政府和市委的坚强领导下，在建设和谐富裕新中卫宏伟目标的激励下，以更加勤勉奉献的精神，以更加执著为民的激情，团结奋发、勇于创新，扎实苦干、追赶跨越，不断迈向新的更大的辉煌！
